# Production of Peroxymonocarbonate by Steady-State Micromolar H_2_O_2_ and Activated Macrophages in the Presence of CO_2_/HCO_3_^–^ Evidenced by Boronate Probes

**DOI:** 10.1021/acs.chemrestox.4c00059

**Published:** 2024-06-25

**Authors:** Edlaine Linares, Divinomar Severino, Daniela R. Truzzi, Natalia Rios, Rafael Radi, Ohara Augusto

**Affiliations:** † Departamento de Bioquímica, Instituto de Química, Universidade de São Paulo, Sao Paulo 05508-900, Brazil; ^‡^Departamento de Bioquímica and ^§^Centro de Investigaciones Biomédicas (CEINBIO), Facultad de Medicina, Universidad de la República, Montevideo 11800, Uruguay

## Abstract

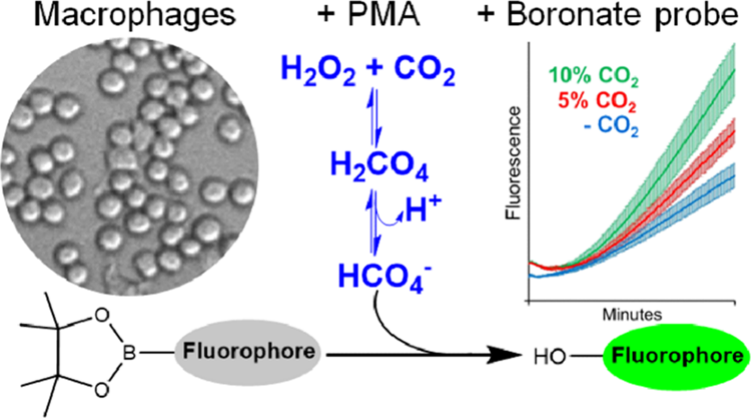

Peroxymonocarbonate (HCO_4_^–^/HOOCO_2_^–^) is produced by the reversible reaction of CO_2_/HCO_3_^–^ with H_2_O_2_ (*K* = 0.33 M^–1^, pH 7.0). Although produced in low yields at physiological pHs and H_2_O_2_ and CO_2_/HCO_3_^–^ concentrations, HCO_4_^–^ oxidizes most nucleophiles with rate constants 10 to 100 times higher than those of H_2_O_2_. Boronate probes are known examples because HCO_4_^–^ reacts with coumarin-7-boronic acid pinacolate ester (CBE) with a rate constant that is approximately 100 times higher than that of H_2_O_2_ and the same holds for fluorescein-boronate (Fl-B) as reported here. Therefore, we tested whether boronate probes could provide evidence for HCO_4_^–^ formation under biologically relevant conditions. Glucose/glucose oxidase/catalase were adjusted to produce low steady-state H_2_O_2_ concentrations (2–18 μM) in Pi buffer at pH 7.4 and 37 °C. Then, CBE (100 μM) was added and fluorescence increase was monitored with time. The results showed that each steady-state H_2_O_2_ concentration reacted more rapidly (∼30%) in the presence of CO_2_/HCO_3_^–^ (25 mM) than in its absence, and the data permitted the calculation of consistent rate constants. Also, RAW 264.7 macrophages were activated with phorbol 12-myristate 13-acetate (PMA) (1 μg/mL) at pH 7.4 and 37 °C to produce a time-dependent H_2_O_2_ concentration (8.0 ± 2.5 μM after 60 min). The media contained 0, 21.6, or 42.2 mM HCO_3_^–^ equilibrated with 0, 5, or 10% CO_2_, respectively. In the presence of CBE or Fl-B (30 μM), a time-dependent increase in the fluorescence of the bulk solution was observed, which was higher in the presence of CO_2_/HCO_3_^–^ in a concentration-dependent manner. The Fl-B samples were also examined by fluorescence microscopy. Our results demonstrated that mammalian cells produce HCO_4_^–^ and boronate probes can evidence and distinguish it from H_2_O_2_ under biologically relevant concentrations of H_2_O_2_ and CO_2_/HCO_3_^–^.

## Introduction

Peroxymonocarbonate (HCO_4_^–^/HOOCO_2_^–^) is a potent oxidant (*E*°^′^ = 1.77 V) produced by the reversible reaction of CO_2_/HCO_3_^–^ with H_2_O_2_ (eq 1) (*K* = 0.33 M^–1^, pH = 7.0).^1^
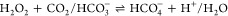
1

Richardson and co-workers extensively studied HCO_4_^–^ formation and properties and first proposed its potential biological relevance supported on chemical grounds. These authors showed that CO_2_ is the component of the CO_2_/HCO_3_^–^ pair that reacts with both H_2_O_2_ and HOO^–^ to produce HCO_4_^–^ (eqs 2–4). Perhydration of CO_2_ forms H_2_CO_4_ (eq 2) which deprotonates (p*K*_a_ = 3.4) (eq 3), and CO_2_ suffers nucleophilic attack by HOO^–^ to produce HCO_4_^–^(eq 4). They also studied these equilibria in detail and showed that HCO_4_^–^ oxidizes organic sulfides,^2^ methionine,^3^ and thiols^4^ more rapidly than H_2_O_2_ does.
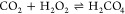
2
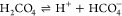
3
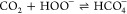
4

Despite the chemical information available on HCO_4_^–^, few researchers investigating biological phenomena related to H_2_O_2_ proposed the involvement of HCO_4_^–^ in them until recently, due to several facts (reviewed in ref (5)). At physiological levels of CO_2_/HCO_3_^–^ and pH, the rate of HCO_4_^–^ formation is quite slow (*k* = 0.034 M^–1^·s^–1^, pH 7.4), and the concentration of HCO_4_^–^ at equilibrium (eq 1) is approximately 1% of that of H_2_O_2_. Although stable in the absence of transition metal ions, HCO_4_^–^ detection in solution was only possible by ^13^C NMR experiments and with concentrations of H_2_O_2_ and CO_2_/HCO_3_^–^ in the molar range.^2,6,7^ Furthermore, most researchers that initially considered the possible involvement of HCO_4_^–^ in biological processes emphasized it either as an oxidant kinetically faster than H_2_O_2_^3,4,7^ or a precursor of CO_3_^•–^.^8−10^ These authors emphasized oxidative damage and pathological processes at a time when redox investigators predominantly focused on redox signaling and cellular responses.

Not surprisingly, the picture started to change after the elegant demonstration that oxidative inactivation of protein tyrosine phosphatase 1B (PTP1B) associated with growth factor stimulation and phosphorylation cascades occurred only when the cells were in medium containing CO_2_/HCO_3_^–^.^11^ Soon after, reports showed that CO_2_/HCO_3_^–^ accelerates hyperoxidation of 2-CysPrxs mediated by H_2_O_2_*in vitro*.^12,13^ And more recently, Winterbourn and co-workers showed that GAPDH oxidation by H_2_O_2_*in vitro* and in cells is greatly stimulated in the presence of CO_2_/HCO_3_^–^.^14^ All of these reports attributed the observed effects to HCO_4_^–^ formation. Indeed, HCO_4_^–^ is a better oxidant than H_2_O_2_ in the heterolytic 2-electron oxidation of thiols because CO_3_^2–^ is a better leaving group than HO^–^. This property accounts for the fact that HCO_4_^–^ oxidizes most nucleophiles with rate constants approximately 10 to 100 times higher than those of H_2_O_2_.^5,15^ However, in the case of PTP1B^16^ and GAPDH,^14^ the increase in rates of catalytic thiol oxidation in the presence of physiological concentrations of CO_2_/HCO_3_^–^ is approximately 3 orders of magnitude higher than in its absence. Certainly, mechanistic details of the latter oxidations remain to be elucidated.^14^ Nevertheless, the fact that HCO_4_^–^ explained the accelerating effects of CO_2_/HCO_3_^–^ on H_2_O_2_-mediated oxidation of thiol proteins involved in cellular responses led to an increased interest in HCO_4_^–^^17^ and in the influence of CO_2_ on mammalian cell metabolism.^18^

In this scenario, the possibility of demonstrating HCO_4_^–^ formation under pathophysiological conditions and in cells became relevant. We previously showed that HCO_4_^–^ oxidizes the boronate probe CBE (coumarin-7-boronic acid pinacolate ester) to the fluorescent COH (7-hydroxycoumarin) with a second-order rate constant that is approximately 2 orders of magnitude higher than that of H_2_O_2_ (*k*_HCO_4_^–^_ = 1.7 × 10^2^ M^–1^ s^–1^; k_H_2_O_2__ = 3.6 M^–1^ s^–1^; pH 7.4 and 37 °C).^5^ Boronate probes initially synthesized to detect H_2_O_2_ soon became the preferential probes to detect peroxynitrite *in vitro* and in cells. Indeed, these probes react with peroxynitrite with second-order rate constants (*k* ∼ 1 × 10^6^ M^–1^ s^–1^) that are much higher than those with H_2_O_2_ (*k*_H_2_O_2__ ∼ 1–2 M^–1^ s^–1^) and with other oxidants (reviewed in ref (19)). Despite the relatively low reactivity of HCO_4_^–^ toward CBE, the absence of straightforward techniques for detecting HCO_4_^–^ at pathophysiological concentrations of H_2_O_2_ and CO_2_/HCO_3_^–^ supports the investigation of boronate probes. In this work, we explore the production of HCO_4_^–^ under steady-state micromolar concentrations of H_2_O_2_ and in RAW 264.7 macrophages activated with PMA with the use of boronate probes.

## Materials and Methods

### Chemicals

All solutions were prepared with ultrapure water purified with a Millipore Milli-Q system. Chemicals and enzymes purchased from commercial sources were DMEM (Sigma D5648), sodium bicarbonate (Sigma S5761), Amplex Red (Invitrogen, Molecular Probes A12222), HRP (Sigma P8375), SOD from bovine erythrocytes (Calbiochem 574594), glucose oxidase (Sigma G2133), glucose (Sigma 8270), catalase (Boehringer 106828), PMA (Sigma P8139), xylenol orange (Sigma 52097), sorbitol (Sigma S1876), ferrous ammonium sulfate (Sigma F3554), sulfuric acid (Merck 100731), DMSO (Sigma 276855), DTPA (Janssen D9390–2), and CBE (Cayman 10818). The synthesis and purification of Fl-B were as previously described.^23^ Stock solutions of boronate probes (CBE or Fl-B) in DMSO protected from light were diluted in the buffer just before use. Hydrogen peroxide was from Synth; solutions were prepared from stock immediately before use, and their concentrations were determined spectrophotometrically by reacting with HRP to produce compound I (Δε_403_ = 5.5 × 10^4^ M^–1^ cm^–1^).^20^

### Generation of Steady-State Concentrations of H_2_O_2_

Micromolar steady-state concentrations of H_2_O_2_ were produced from glucose oxidation by glucose oxidase (GO) in the presence of catalase as previously described.^21^ In summary, glucose (10 mM) in phosphate buffer (100 mM) containing DTPA (0.1 mM) at pH 7.4 and 37 °C was incubated for 15 min before the addition of GO to produce H_2_O_2_ at rates varying from 0.2 to 2.0 μM/min. After 5 min, we added catalase amounts sufficient to produce steady-state concentrations of H_2_O_2_ varying from approximately 2 to 20 μM. The concentrations of H_2_O_2_ produced were quantitated with the FOX reagent.^22^ At different incubation times, sample aliquots were mixed (1:1 v/v) with FOX reagent (212 μM xylenol orange, 520 μM Fe^2+^, 106 mM H_2_SO_4_, and 215 mM sorbitol) in a 96-well plate at 37 °C. After 40 min, measurements of the absorbance at 560 nm in a microplate reader (Infinite M200, Tecan) permitted the calculation of H_2_O_2_ concentrations from a calibration curve obtained with known H_2_O_2_ concentrations.

### Kinetics of CBE Oxidation by Steady-State Concentrations of H_2_O_2_ in the Absence and Presence of CO_2_/HCO_3_^–^

After establishing the conditions to generate a low micromolar steady state of H_2_O_2_ concentrations as described above, we performed kinetic studies of CBE oxidation by these H_2_O_2_ concentrations in the absence and presence of CO_2_/HCO_3_^–^(25 mM). Fifteen minutes after catalase addition, we added CBE (100 μM) to each steady-state H_2_O_2_ concentration and monitored CBE oxidation by measuring fluorescence (λ_exc_ = 332 nm, λ_em_= 456 nm) in a microplate reader (Infinite M200, Tecan) at 37 °C. The samples containing CO_2_/HCO_3_^–^ (25 mM) were maintained under a gaseous atmosphere of 5% CO_2_/air throughout the experiments. To determine the second-order rate constants of the reaction of CBE with H_2_O_2_ or HCO_4_^–^ by the initial rate approach, we converted the Δfluorescence values to the concentration of oxidized CBE (see the Results Section).

### Determination of the Second-Order Rate Constant of the Oxidation of Fl-B by HCO_4_^–^

The kinetics of Fl-B oxidation by H_2_O_2_ and HCO_4_^–^ were monitored in a stopped-flow spectrophotometer (SX20 Applied Photophysics) by measurements of the fluorescence of Fl-B oxidized product (λ_exc_ = 492 nm and total emission). In the case of H_2_O_2_ kinetics, we followed the classical protocol with millimolar concentrations of H_2_O_2_ due to the small rate constant of the reaction (*k* = (1.72 ± 0.2) M^–1^·s^–1^).^23^ Fl-B (4 μM) in phosphate buffer (100 mM) containing DTPA (0.1 mM), pH 7.4, was mixed (1:1, v/v) with different concentrations of H_2_O_2_ (5–20 mM) in the same buffer. The final concentration of Fl-B was 2 μM and that of H_2_O_2_ varied from 2.5 to 10 mM. In the case of HCO_4_^–^ kinetics, we added additional procedures to the usual protocol. Thus, the stock solutions of CO_2_/HCO_3_^–^ were prepared in phosphate buffer (100 mM) containing DTPA (0.1 mM), pH 7.4, and maintained in a container sealed with rubber. CO_2_/HCO_3_^–^ solutions were preincubated with H_2_O_2_ solutions for 10 min to permit equilibration and HCO_4_^–^ formation in a sealed container. Gas tight syringes were used throughout the experiments. Fl-B (4 μM) in phosphate buffer (100 mM) containing DTPA (0.1 mM), pH 7.4, was mixed (1:1, v/v) with H_2_O_2_ (2.5 mM) and CO_2_/HCO_3_^–^ (0–100 mM) in the same buffer. The final pH of the mixtures was controlled to be in the range of (7.4 ± 0.1), and the temperature was 37 °C. The obtained kinetic curves fitted to single exponential equations provided the corresponding *k*_obs_ values. These values plotted against H_2_O_2_ or HCO_4_^–^ concentrations resulted in straight lines, the slope of which provided the second-order rate constants of the reactions.

### Cell Cultures

Raw macrophage cells (264.7 cell line) obtained from BCRJ (Cell Bank from Rio de Janeiro, Brazil) were cultured in DMEM supplemented with 10% fetal bovine serum, 44 mM sodium bicarbonate, 0.1 g/L streptomycin, and 0.025 g/L ampicillin maintained in a humidified atmosphere with 5% CO_2_ at 37 °C. Before treatments, cells (5 × 10^4^ cells/well) were plated in 96-well plates and maintained under the same conditions for 72 h. Confluent cells washed 3 times received media before the experiments performed at pH 7.4 and 37 °C (total volume of 200 μL). The media were modified DPBSG (Na_2_HPO_4_ (21.3 mM), KH_2_PO_4_ (3.87 mM), KCl (2.67 mM), NaCl (138 mM), MgCl_2_ (0.49 mM), CaCl_2_ (0.88 mM), glucose (5.5 mM), and DTPA (0.1 mM), pH 7.4, containing 0, 21.6, or 42.2 mM HCO_3_^–^ equilibrated with 0, 5, or 10% CO_2_,^11,24^ respectively). For simplicity, these concentrations of CO_2_/HCO_3_^–^ were abbreviated as 5 and 10% CO_2_, respectively, in the notations to differentiate the curves in all figures.

### Determination of H_2_O_2_ Concentrations Produced by RAW Macrophage Activated with PMA

The production of H_2_O_2_ by macrophage cells activated with PMA was monitored by measurements of resorufin produced from the oxidation of Amplex Red by HRP/H_2_O_2_ in the presence of SOD.^25^ Washed confluent cells in modified DPBSG in the absence or presence of CO_2_/HCO_3_^–^, as described above, were incubated with Amplex Red (50 μM), HRP (1U/mL), and SOD (50 μg/mL) for 10 min at 37 °C. Then, the cells were activated with PMA (1 μg/mL), and the kinetics of H_2_O_2_ production were observed, followed by resorufin fluorescence (λ_exc_ = 535 nm, λ_em_ = 595 nm), in a microplate reader (Synergy H1, BioTek) with a gas controller (BioTek) to maintain 0, 5, or 10% CO_2_, depending on the experiment. Catalase (250 U/mL) was added in some experiments prior to PMA activation. The concentration of produced H_2_O_2_ was calculated using a standard curve of resorufin formed by the oxidation of Amplex Red by HRP and known concentrations of H_2_O_2_.

### Cell Viability Measurements

Cells were plated in 12-well plates (2.5 × 10^5^ cells/well) 72 h before PMA activation. Washed confluent cells in modified DPBSG (1 mL) in the absence or presence of CO_2_/HCO_3_^–^ were incubated and activated with PMA (1 μg/mL) as described above. Control cells did not receive PMA. After 1 h incubation, washed cells were harvested into 1 mL of PBS. Cell suspension aliquots were diluted (10×) into Muse reagent (Muse Cell Count and Viability Assay), incubated for 5 min, and analyzed in a cell analyzer (Muse cell analyzer, Millipore). Viable cells are expressed as the percentage of counted cells (2.0 × 10^3^).

### Nitrite Measurements

To verify eventual NO^•^ production by macrophages activated by PMA under our experimental conditions, we determined the levels of NO_2_^–^ in the supernatants of the cells using the Griess method. Washed confluent cells in modified DPBSG were incubated and activated with PMA (1 μg/L) as described above; control cells did not receive PMA. After 1 h incubation, the supernatant of the cells was centrifuged for 5 min at 1500 rpm. Aliquots of the supernatant were mixed with a Griess reagent (1:1) in a 96-well plate. After 15 min at room temperature, the absorbance at 540 nm was measured using a microplate reader (Infinite M200, Tecan). Nitrite concentration was calculated from a standard curve obtained under the same experimental conditions using a Griess reagent and standard NO_2_^–^ solutions. To have a positive control of macrophages producing NO^•^, we performed parallel experiments with macrophages incubated for 20 h with culture medium containing INF-λ (200 U/mL) and LPS (1 μg/mL) before washing and activating with PMA. The culture media of these cells and the cells not pretreated with INF/LPS were collected for NO_2_^–^ determination. The cells pretreated with INF/LPS were washed, transferred to DPBSG, activated with PMA, and treated as above for NO_2_^–^ determination.

### Boronate Probe Oxidation by PMA-Activated Macrophages in the Absence and Presence of CO_2_/HCO_3_^–^

Washed confluent cells in modified DPBSG in the absence or presence of CO_2_/HCO_3_^–^ as described above were incubated with a 30 μM boronate probe (CBE or Fl-B) for 10 min at 37 °C in a microplate reader (Synergy H1, BioTek) with a gas controller (BioTek) installed to maintain 0, 5, or 10% CO_2_, respectively. Then, the cells were activated by PMA (1 μg/mL), and the kinetics of the oxidation of boronate probes were observed, followed by fluorescence of the CBE (λ_exc_ = 332 nm, λ_em_ = 456 nm) or the Fl-B oxidation product (λ_exc_ = 492 nm, λ_em_ = 520 nm).

## Results

### CBE Detects HCO_4_^–^ Produced from Low Micromolar Steady-State Concentrations of H_2_O_2_ in the Presence of Physiological Levels of CO_2_/HCO_3_^–^

**Figure 1:**
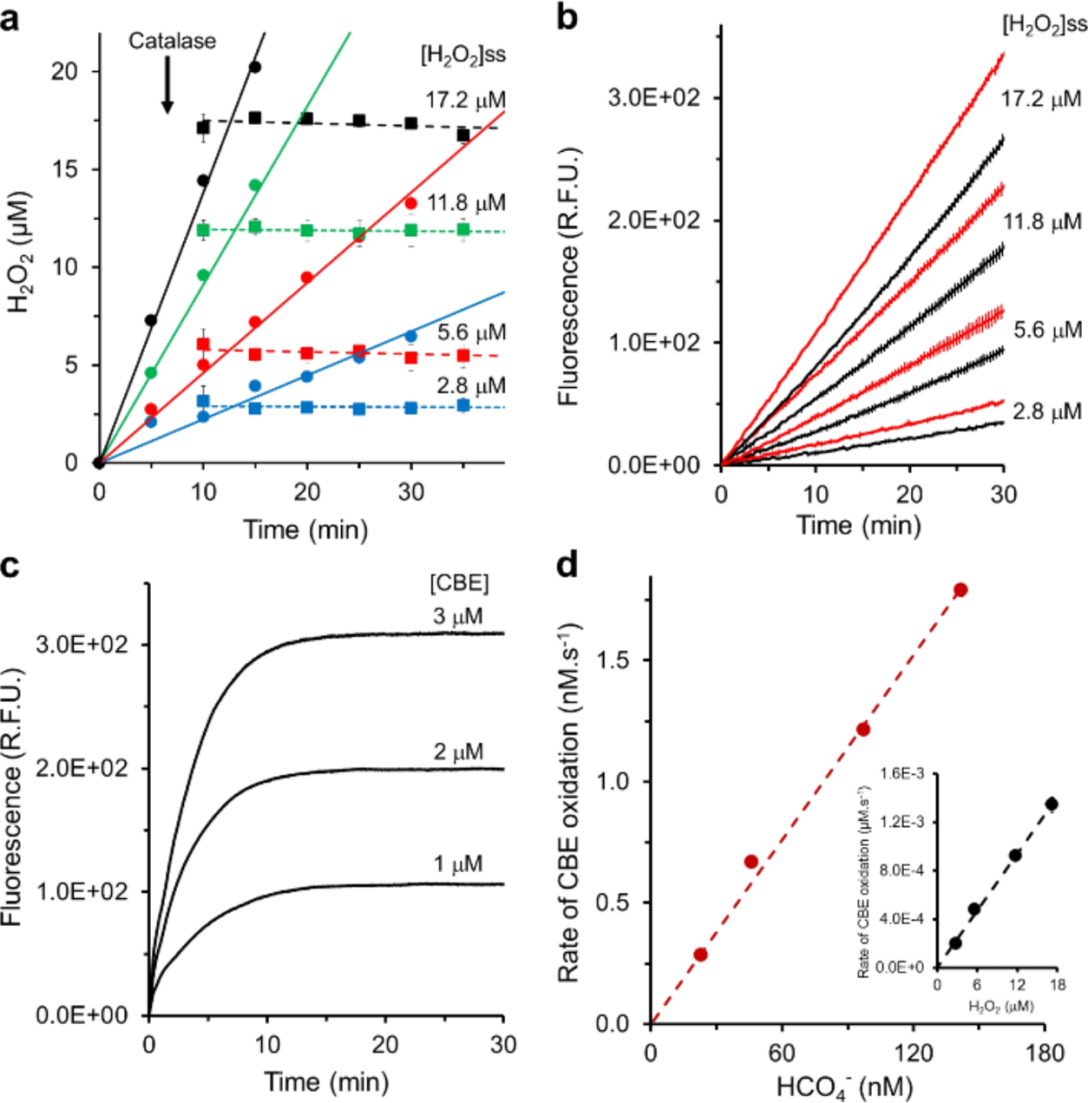
Boronate probe CBE detects HCO_4_^–^ formation from low micromolar steady-state concentrations of H_2_O_2_ in the presence of CO_2_/HCO_3_^–^ (25 mM). (a) Production of H_2_O_2_ at different rates by the oxidation of glucose (10 mM) and adjusted concentrations of glucose oxidase before (full lines, circles) or 5 min after catalase addition (interrupted lines, squares); the attained steady-state H_2_O_2_ concentrations are displayed in the figure. (b) Kinetics of CBE (100 μM) oxidation by the specified steady-state concentrations of H_2_O_2_ obtained as in (a) in the absence (black lines) or presence (red lines) of CO_2_/HCO_3_^–^ (25 mM). (c) Kinetic profile of complete oxidation of the specified concentrations of CBE by excess H_2_O_2_ (5 mM). (d) Plots of CBE oxidation rates from the experiments shown in 1B expressed in nM·s^–1^ or μM·s^–1^ against the concentration of HCO_4_^–^ or H_2_O_2_, respectively (inset).

We used glucose (10 mM) and adjusted amounts of glucose oxidase and catalase to obtain low micromolar steady-state concentrations of H_2_O_2_ as described in the Materials and Methods Section.^21^Figure 1a displays the produced concentrations of H_2_O_2_ measured by the FOX method before (circles, full lines) or 5 min after addition of adjusted catalase concentrations (squares; interrupted lines) to permit production of low micromolar steady-state concentrations of H_2_O_2_ as specified. In parallel experiments, the same steady-state concentrations of H_2_O_2_ were established, and 15 min after catalase addition, CBE (100 μM) was added and its reaction with H_2_O_2_ was monitored by fluorescence increase due to the formation of the oxidized fluorescent product COH (Figure 1b, black lines). As expected, CBE reacted in a time-dependent and H_2_O_2_ concentration-dependent manner. Then, the same experiments were repeated in the presence of CO_2_/HCO_3_^–^ (25 mM) (Figure 1b, red lines). It was observed that each of the steady-state concentrations of H_2_O_2_ reacted with CBE more rapidly (roughly about 30%) in the presence of CO_2_/HCO_3_^–^ (25 mM) than in its absence. These results are consistent with the formation of steady-state HCO_4_^–^ concentrations directly proportional to the steady-state concentrations of H_2_O_2_ as expected from eq 1 and the equation derived from it (eq 5).
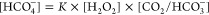
5where *K* is 0.33 M^–1^; [CO_2_/HCO_3_^–^] is 25 mM; [H_2_O_2_] is each measured steady-state concentration of H_2_O_2_.

If the above assumption is reasonable, we should be able to calculate the second-order rate constant of the reaction of CBE with both H_2_O_2_ and HCO_4_^–^ and find values close to those previously reported using stopped-flow kinetics and the pseudo-first-order approach.^5^ Under the experimental conditions of Figure 1b, the second-order rate constants can be obtained by the initial rate approach after conversion of the ΔFluorescence values displayed in Figure 1b to the concentration of oxidized CBE (COH). The conversion factor was determined by oxidizing 1, 2, and 3 μM CBE with a high excess of H_2_O_2_ (5 mM) up to complete CBE oxidation (Figure 1c). The average ΔF value that accounted for 1 μM CBE oxidation was 103 ± 8.0 au. The latter value permitted the calculation of the initial rate of CBE oxidation in nM·s^–1^ or μM·s^–1^ at each of the conditions displayed in Figure 1b. The obtained values were plotted against the steady-state concentrations of H_2_O_2_ (measured by the Fox method) (Figure 1d, inset) or HCO_4_^–^ (calculated from eq 5) (Figure 1d). The slopes of the obtained straight lines divided by the CBE concentration (100 μM) provided the second-order rate constants for the reaction of CBE with H_2_O_2_ as 0.8 M^–1^ s^–1^ and with HCO_4_^–^ as 1.3 × 10^2^ M^–1^ s^–1^ (Figure 1d) in the range of the values previously reported for coumarin-derived probes.^5,19^ Taken together, these results (Figure 1) show that low micromolar concentrations of H_2_O_2_ in the presence of physiological concentrations of HCO_3_^–^/CO_2_ sustain steady-state concentrations of HCO_4_^–^ that are distinguishable from H_2_O_2_ by the increased rate of CBE oxidation. It is important to note that the rate of oxidation of a compound by H_2_O_2_ in the presence of CO_2_/HCO_3_^–^ is a combination of oxidation due to both H_2_O_2_ and equilibrated HCO_4_^–^.^3−5^

### Second-Order Rate Constant of Fl-B Oxidation by H_2_O_2_ and HCO_4_^–^ at pH 7.4 and 37 °C

CBE is useful in *in vitro* studies (Figure 1), but the spectroscopic characteristics of its oxidation product (λ_ex_ = 332 nm; λ_em_ = 450 nm) can limit cell studies by methodologies other than those using plate readers. Therefore, we next determined the second-order rate constant of the Fl-B reaction with HCO_4_^–^ at pH 7.4 and 37 °C. The second-order rate constants of the Fl-B reaction with H_2_O_2_ at 37 °C ((1.72 ± 0.20) M^–1^·s^–1^) were previously determined.^23,26^

**Figure 2:**
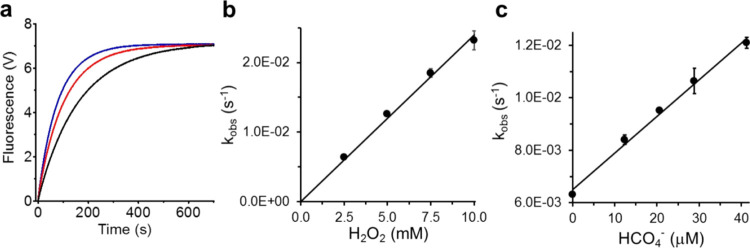
Kinetics of Fl-B oxidation by H_2_O_2_ and HCO_4_^–^. (a) Representative kinetic curves of Fl-B oxidation (2 μM) by H_2_O_2_ (2.5 mM) in phosphate buffer (100 mM) containing DTPA (0.1 mM), pH 7.4, 37 °C, in the absence (black trace) or the presence of CO_2_/HCO_3_^–^ 25 mM (red trace) or 50 mM (blue trace). (b) Determination of the second-order rate constant of the reaction of Fl-B with H_2_O_2_. (c) The same as (b) for determination of the second-order rate constant of the reaction of Fl-B with HCO_4_^–^. The plotted values in panels b and (c) are the mean ± SD obtained from three independent experiments.

The kinetics of Fl-B oxidation were monitored by the intrinsic fluorescence of the Fl-B oxidation product, fluorescein (Fl) in phosphate buffer (100 mM) containing DTPA, pH 7.4 at 37 °C. Representative kinetics of the oxidation of Fl-B (2 μM) by H_2_O_2_ (2.5 mM) in the absence (black trace) and in the presence of CO_2_/HCO_3_^–^ 25 mM (red trace) or 50 mM (blue trace) are displayed in Figure 2a. These experiments were repeated first with different concentrations of H_2_O_2_ (2.5–10 mM), maintaining the Fl-B concentration constant. The kinetic curves obtained for each H_2_O_2_ concentration fitted to single exponential curves provided the corresponding *k*_obs_ values. These values plotted against the employed H_2_O_2_ concentrations provided a straight line, the slope of which equals the second-order rate constant for the reaction of Fl-B with H_2_O_2_ as 2.4 ± 0.1 M^–1^ s^–1^ (Figure 2B), in agreement with the previously reported value.^23,26^

Next, we repeated the experiments shown in Figure 2a, maintaining the Fl-B concentration (2 μM, final concentration) and mixing it with H_2_O_2_ (2.5 mM, final concentration) preincubated with different concentrations of CO_2_/HCO_3_^–^ (15, 25, 35, and 50 mM). Treatment of the obtained kinetic curves provided the corresponding *k*_obs_ values and the value of the second-order rate constant for the reaction of Fl-B with HCO_4_^–^ as (1.39 ± 0.06) x10^2^ M^–1^·s^–1^ at pH 7.4 and 37 °C (Figure 2c). As expected, the line resulting from the *k*_obs_*versus* concentration did not intersect the *y* axis at zero but at the *k*_obs_ value of H_2_O_2_ 2.5 mM because of H_2_O_2_ contribution to Fl-B oxidation by HCO_4_^–^.^3−5^

### RAW 264.7 Macrophages Activated with PMA: Characterization of Oxidant Production

To examine whether boronate probes can distinguish HCO_4_^–^ from H_2_O_2_ in cells, we selected macrophages (RAW 264.7) and activated them exclusively with PMA to preclude, or decrease to a minimum, the production of reactive species other than O_2_^•–^ and H_2_O_2_.^27^ Particularly relevant to avoid were those reactive species that react with boronate probes more rapidly than HCO_4_^–^, such as peroxynitrite (*k* ∼ 1 × 10^6^ M^–1^ s^–1^)^28^ and HOCl (*k*_Fl-B_= 6.2 x10^2^ M^–1^ s^–1^, pH 7.4, 37 °C).^23^ Biological production of HOCl requires the enzyme myeloperoxidase (MPO) and H_2_O_2_,^29^ but RAW macrophages express quite low levels of MPO.^30,31^ On the other hand, RAW macrophages exclusively activated with PMA^27^ are unlikely to produce considerable levels of NO^•^ and, consequently, of peroxynitrite.^32^

**Figure 3:**
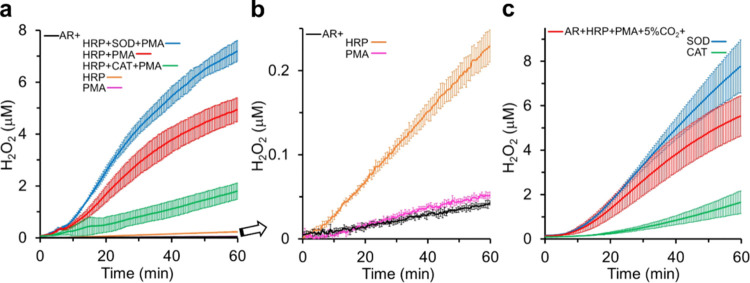
Production of H_2_O_2_ by confluent RAW macrophages activated by PMA monitored by Amplex Red oxidation. (a) Representative kinetic curves of H_2_O_2_ production by confluent macrophages in DPBSG, pH 7.4, 37 °C containing 50 μM Amplex Red (AR) and with or without PMA (1 μg/mL), HRP (1 U/mL), SOD (50 μg/mL), and catalase (250 U/mL) as specified; the controls are also displayed but hardly visible. (b) Replot of the controls in (a) in an amplified scale. (c) Parallel experiments to evaluate CO_2_/HCO_3_^–^ effects on H_2_O_2_ production by PMA-activated macrophages. Experimental conditions are the same as (a) except for the presence of 21.6 mM HCO_3_^–^ equilibrated with 5% CO_2_ abbreviated as 5% CO_2_ in the figure. The plotted values are the mean ± SD of three repetitions of each experiment.

Confluent RAW macrophages activated with PMA (1 μg/mL) in DPBSG (pH 7.4, 37 °C) produced extracellular H_2_O_2_, which was quantitated by the HRP-catalyzed oxidation of Amplex Red (AR) (50 μM) to resorufin in the presence of SOD (50 μg/mL) (Figure 3A). In the absence of SOD, the detected H_2_O_2_ concentrations were lower as expected.^25^ In contrast to SOD, catalase strongly inhibited Amplex Red oxidation because it depends on H_2_O_2_ produced by PMA-activated macrophages and added HRP (Figure 3a). Considerable levels of H_2_O_2_ occurred only in the case of cells activated with PMA and in the presence of both HRP and Amplex Red. This point becomes clearer in Figure 3b, which amplifies the scale of the H_2_O_2_ concentration produced in the control experiments of Figure 3a (cells + Amplex Red; cells + Amplex Red + PMA; cells + Amplex Red + HRP). The marginal levels of H_2_O_2_ detected in the absence of HRP (Figure 3a,b) confirmed the low levels of MPO in RAW macrophages.^30,31^ In parallel, these results excluded the production of considerable levels of HOCl under the experimental conditions employed (Figure 3a,b).

The concentrations of extracellular H_2_O_2_ produced by PMA-activated macrophages increased some with PMA concentration (0.5 to 2.0 μg/mL), but not considerably (data not shown). Therefore, we used 1 μg/mL PMA in all of the experiments reported here. Under these conditions, the average concentration of H_2_O_2_ obtained after 60 min incubation from numerous independent experiments was approximately 8.0 ± 2.5 μM. It is difficult to compare our values with those in the literature because most studies using RAW macrophages activated with PMA do not report quantitative data for H_2_O_2_ production and/or rely on methodologies less specific than the Amplex Red assay.^33^ The presence of CO_2_/HCO_3_^–^ marginally affected the levels of H_2_O_2_ produced by activated macrophages (Figure 3c).

**Figure 4:**
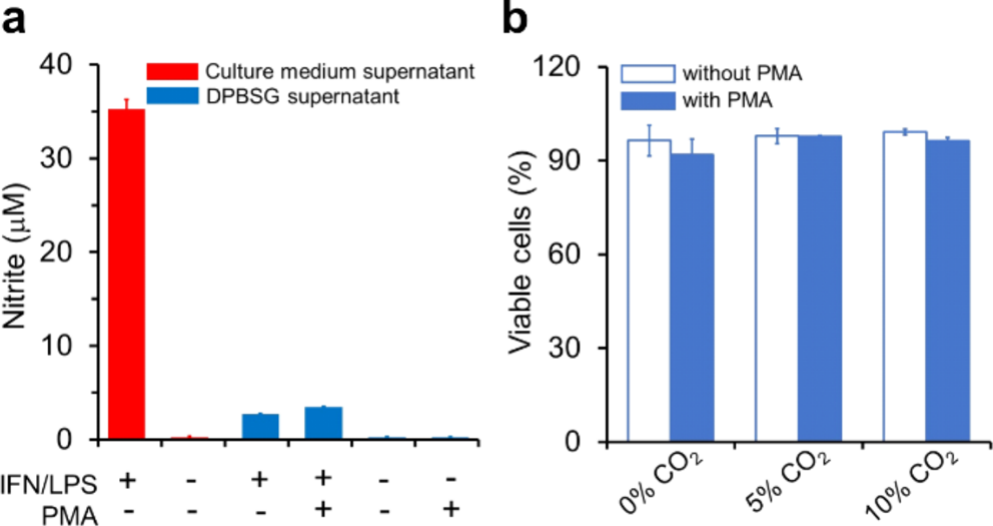
RAW macrophages activated by PMA do not produce NO^•^ and maintain viability. (a) NO^•^ production was evaluated by measuring NO_2_^–^ levels in the supernatants of RAW macrophages under our usual experimental conditions in parallel with that of a positive control. The levels of NO_2_^–^ before washing (culture media) or after washing, transfer to DPBSG, and activation or not with PMA (1 μg/mL) are displayed as specified. (b) Cell viability monitored by the Muse Cell Count and Viability Assay in the Muse cell analyzer. Viable cells were expressed as the percentage of counted cells (2.0 × 10^3^). The plotted values are the mean ± SD obtained from three repetitions.

Next, we confirmed that under the tested experimental conditions, macrophages activated with PMA do not produce sufficient levels of NO^•^ to react with the formed O_2_^•–^ to produce peroxynitrite.^32^ To this end, we determined the levels of NO_2_^–^ by the Griess assay in the supernatants of RAW macrophages activated or not with PMA in parallel with that of positive controls, that is, macrophages pretreated with INF-λ (200 U/mL) and LPS (1 μg/mL) in culture medium before PMA activation. As shown in Figure 4a, the culture medium supernatant of macrophages pretreated with INF-λ/LPS contained considerable levels of NO_2_^–^ (35.3 ± 1.0 μM) in contrast to those of untreated macrophages (Figure 4a, red bars). The DPBSG supernatants of the cells pretreated with INF-λ/LPS after washing, transfer to media, activation with PMA, and 1 h incubation contained levels of NO_2_^–^ that were quite lower (3.5 ± 0.1 μM) and not much different from those of pretreated cells not activated with PMA (2.7 ± 0.1 μM) (Figure 4a, blue bars). The supernatants of the cells activated exclusively with PMA contained undetectable levels of NO_2_^–^, excluding the production of NO^•^ and thus, of peroxynitrite, under the employed experimental conditions (Figure 4a).

We also examined whether macrophages activated or not with PMA and submitted to 1 h long incubation under 0, 5, or 10% CO_2_ maintained their viability by using the Muse Count and Viability Assay (see Materials and Methods Section). Most cells remained viable under all tested conditions, as shown by the percentage of viable cells among the counted cells (2.0 × 10^3^) (Figure 4b).

### Boronate Probes Can Distinguish HCO_4_^–^ from H_2_O_2_ Released by RAW 264.7 Macrophages Activated with PMA

**Figure 5:**
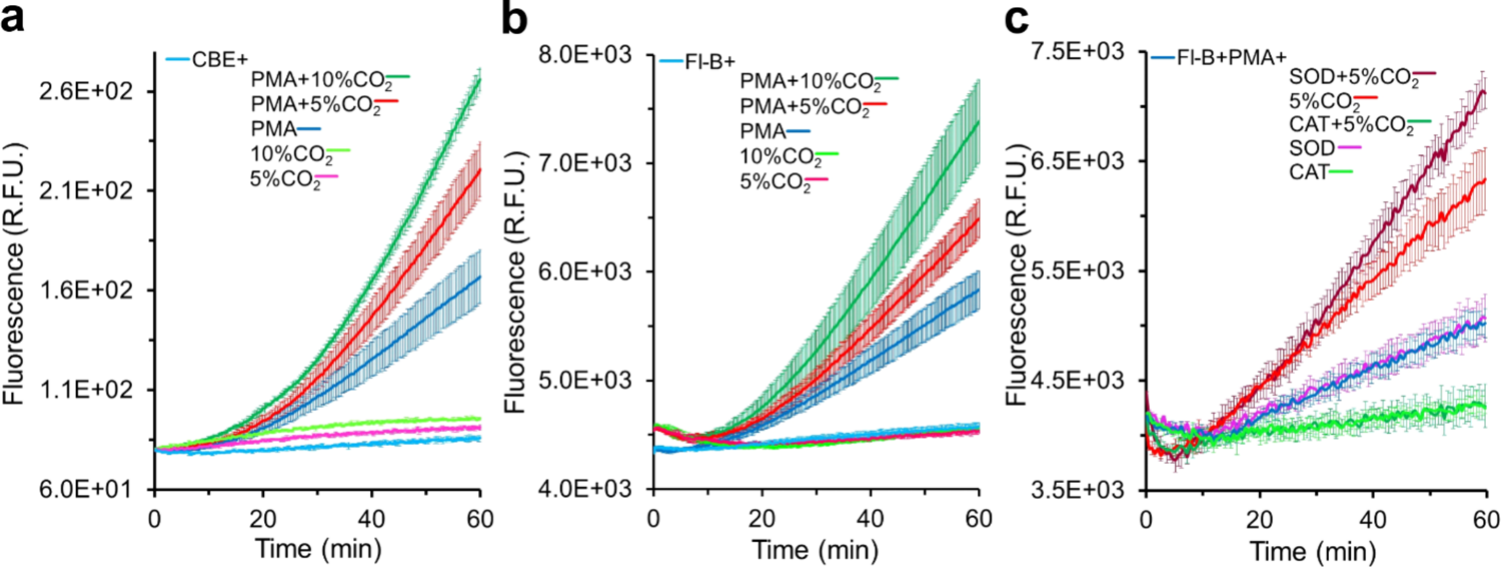
CBE and Fl-B distinguish HCO_4_^–^ from H_2_O_2_ in PMA-activated RAW macrophages producing H_2_O_2_ in the presence of CO_2_/HCO_3_^–^. (a) Kinetics of CBE oxidation by confluent RAW macrophage cells in DPBSG containing 30 μM CBE in the absence or presence of CO_2_/HCO_3_^–^ at the specified concentrations incubated for 10 min at 37 °C before activation or with PMA (1 μg/mL). The same as (a) with substitution of CBE by Fl-B. (c) Kinetics of Fl-B oxidation by confluent RAW macrophages in DPBSG containing 30 μM Fl-B (and also SOD (50 μg/mL) or catalase (250 U/mL) when specified) in the absence or presence of CO_2_/HCO_3_^–^ (5%/21.6 mM) activated with PMA. All of the plotted values are the mean ± SD obtained from three repetitions of each experiment.

Next, we tested whether the boronate probes CBE and Fl-B could evidence HCO_4_^–^ formation by PMA-activated macrophages. Confluent macrophages in modified DPBSG in the absence or presence of CO_2_/HCO_3_^–^ were incubated with 30 μM CBE for 10 min at 37 °C in a microplate reader maintained at 0, 5, or 10% CO_2_ (see Materials and Methods Section). Then, PMA (1 μg/mL) was added, and the kinetics of the oxidation of CBE were observed, followed by fluorescence. In the absence of CO_2_/HCO_3_^–^, time-dependent oxidation of CBE was observed (Figure 5a, blue curve). The kinetic profile of CBE oxidation was consistent with that obtained for H_2_O_2_ production (Figure 3a) since the rate of CBE oxidation by H_2_O_2_ is considerably lower than the rate of the oxidation of Amplex Red by H_2_O_2_/HRP.^34^ Relevantly, the rate of CBE oxidation increased in the presence of CO_2_/HCO_3_^–^ and the increase was roughly proportional to the pair concentration ((21.6 mM HCO_3_^–^ equilibrated with 5% CO_2_) (Figure 5a, red curve) and (42.2 mM HCO_3_^–^ equilibrated with 10% CO_2_) (Figure 5a, green curve)). We abbreviated these CO_2_/HCO_3_^–^ concentrations as 5 and 10% CO_2_, respectively, in the notations to differentiate the curves in all figures for the sake of simplicity.

Substitution of CBE by Fl-B led to results that were qualitatively similar (Figure 5b) and consistent with different properties of their respective oxidation products. Indeed, the Fl-B oxidation product fluorescein (Fl) has a higher fluorescence quantum yield than COH,^23^ justifying the relatively higher fluorescence values obtained with Fl-B (compare Figure 5a with b). Nevertheless, the kinetic profiles of the oxidation of both boronate probes were quite similar under all of the tested conditions. These results indicated that in the presence of CO_2_/HCO_3_^–^, part of the H_2_O_2_ being produced by PMA-activated macrophages (Figure 3a) reacts with dissolved CO_2_ to produce HCO_4_^–^ (eqs 1–4), leading to increased rates of CBE or Fl-B oxidation in a manner dependent on CO_2_/HCO_3_^–^ concentration (Figure 5a,b). In agreement, the presence of CO_2_/HCO_3_^–^ marginally affected the production of H_2_O_2_ by activated macrophages (Figure 3c), whereas catalase addition abolished the stimulatory effect of CO_2_/HCO_3_^–^ on the oxidation of the probes (see, for instance, Figure 5c, green curve).

The above results (Figure 5) showed that boronate probes can evidence HCO_4_^–^ formation from PMA-activated macrophages, distinguishing it from H_2_O_2_ in the bulk solution or, in other words, in the extracellular environment.

### Fl Detection in Resting and PMA-Activated Macrophages in the Presence of Fl-B

Although PMA-activated macrophages produce mainly extracellular H_2_O_2_, this small and uncharged oxidant can transverse biological membranes and aquaporins (peroxiporins) facilitate its transport into cells.^35−37^ Likewise, CO_2_ is membrane permeable and continuously produced by cells, whereas HCO_3_^–^ has a family of transporters, including exchangers and cotransporters;^11,18,38^ eventual HCO_4_^–^ exchange and/or transport remain uninvestigated. Thus, it was interesting to examine the possibility that boronate probes can detect intracellular oxidants produced from resting and PMA-activated macrophages by fluorescence microscopy. The selected probe was Fl-B because of the spectroscopic properties of its oxidation product^23^ and because it is a neutral molecule capable of traversing cellular membranes through diffusion. Conversely, in aqueous solution and at pH 7.4, the predominant species of Fl is the dianionic form (p*K*_a_ 4.3 and 6.4) in equilibrium with a small proportion of the monoanionic and neutral forms.^23,39^ The obtained results did not expand the information obtained up to this point but are presented in the Supporting Information (Figures S1 and S2) because they provided useful clues for future studies of HCO_4_^–^ in cells.

## Discussion

The recognition of HCO_4_^–^ as a relevant biological oxidant is just beginning but may have significant implications for human health. HCO_4_^–^ may play a role in cellular responses to H_2_O_2_ and redox signaling by oxidizing specific thiol proteins with extremely high efficiency as compared to H_2_O_2_.^14^ HCO_4_^–^ may also participate in oxidative damage because it oxidizes Met and Cys protein residues about 100 times faster than H_2_O_2_^3,4,7^ and HCO_4_^–^ reduction by transition metal ions and metalloproteins can produce the reactive CO_3_^•–^.^5,8−10^ To advance the understanding of HCO_4_^–^ biochemistry, it is important to count on simple and widespread methodologies to detect it. However, there were no tested methodologies to evidence HCO_4_^–^ formation even under pathophysiological concentrations of H_2_O_2_ (about 1–10 μM intracellularly).^36^ Our work showed that boronate probes distinguish HCO_4_^–^ from H_2_O_2_ under such conditions.

Our results demonstrated that low micromolar steady-state concentrations of H_2_O_2_ in the presence of physiological concentrations of CO_2_/HCO_3_^–^ produce steady-state concentrations of HCO_4_^–^ in the nanomolar range that are distinguishable from H_2_O_2_ by the increased rate of CBE oxidation (Figure 1). This can be extended to all boronate probes because they react with H_2_O_2_ and HCO_4_^–^ by the same mechanism and with similar second-order rate constants (*k*_H_2_O_2__ ∼ 1 M^–1^ s^–1^; *k*_HCO_4_^–^_ ∼ 10^2^ M^–1^ s^–1^).^19,40^ The overall results displayed in Figure 1 showed that boronate probes evidence HCO_4_^–^ formation under pathophysiological concentrations of H_2_O_2_ and CO_2_/HCO_3_^–^, particularly when we compare the rate of boronate probe oxidation in the absence and presence of CO_2_/HCO_3_^–^. One-time point measurement of the fluorescence in both of these conditions is not ideal since the expected fluorescence differences are small (Figure 1b) due to the low concentration of HCO_4_^–^, as compared to H_2_O_2_ at equilibrium (eq 5),^5^ and to the relatively small second-order rate constant value of the reaction of HCO_4_^–^ with boronate probes.^19^

We also tested CBE and Fl-B in the cells. Since these experiments were the first in cell cultures to the best of our knowledge, we selected macrophages (RAW 264.7) activated exclusively with PMA to limit or preclude the formation of oxidants other than O_2_^•–^ and H_2_O_2_. We confirmed and quantified the formation of H_2_O_2_ by macrophages activated with PMA (Figure 3a) and excluded the possibility of concomitant formation of significant concentrations of HOCl (Figure 3b) and peroxynitrite (Figure 4a) as emphasized above. We also showed that CO_2_/HCO_3_^–^ marginally affected the concentration of H_2_O_2_ formed (Figure 3c) and that the cells remained viable up to 1 h of incubation (Figure 4b). As could be expected from the results obtained with steady-state concentrations of H_2_O_2_, cells continuously producing extracellular H_2_O_2_ also continuously produce HCO_4_^–^ when in the presence of CO_2_/HCO_3_^–^, as revealed by the increased rates of oxidation of both CBE (Figure 5a) and Fl-B (Figure 5b). The rates of probe oxidation increased with the concentration of CO_2_/HCO_3_^–^ for the same H_2_O_2_ concentration, supporting HCO_4_^–^ formation. Likewise, the fact that catalase addition completely abolished the stimulatory effect of CO_2_/HCO_3_^–^ on boronate probe oxidation (see, for instance, Figure 5c) confirmed HCO_4_^–^ formation.

Despite PMA-activated macrophages producing mostly extracellular H_2_O_2_, we also examined resting and activated cells incubated for 60 min with Fl-B under our experimental conditions by fluorescence microscopy (Figures S1 and S2). These experiments did not provide additional insights to the other experiments but rendered useful information for future studies of HCO_4_^–^ in cells. Even through structural modifications, boronate probes are unlikely to acquire enough sensibility to detect HCO_4_^–^ formed from basal H_2_O_2_ concentrations (Figure S2). The considerable variability of fluorescence intensity obtained for the same sample in different microscopy experiments (Figures S1 and S2) can likely be attributed to the change in the atmosphere of the cells prior to imaging. These results argue once again for importance of performing cell culture experiments under physiologically relevant tensions of O_2_ and CO_2_.^18,41,42^ The use of such conditions throughout the experiments will likely be important to monitor intracellular production of HCO_4_^–^ from agents that produce H_2_O_2_ concentrations for redox signaling, such as growth factors^11,43^ and from compounds that suffer redox-cyling.^44,45^ Similarly, such conditions will be important to carry out experiments with cells that produce extracellular H_2_O_2_ more rapidly and at higher levels than RAW macrophages activated with PMA (Figure 3) and in the presence of boronate probes designed for intracellular accumulation and retention.^19^ The latter experiments may provide information about the existence of possible transporters that allow HCO_4_^–^ entry into cells.

In conclusion, our work demonstrated that boronate probes are important molecular tools to detect HCO_4_^–^ and distinguish it from H_2_O_2_ under pathophysiological concentrations of H_2_O_2_. These demonstrations may help to substantiate HCO_4_^–^ as a relevant biological oxidant.^5,11,14,17,18^ From a toxicological perspective, it is important to note that the levels of CO_2_ are increasing in the atmosphere, and accumulating evidence indicate that environmentally relevant elevations in CO_2_ (<5000 ppm) may pose direct risks to human health.^46^ The mechanisms remain poorly understood and are certainly not exclusively redox mechanisms. In addition to react with biologically ubiquitous oxygen metabolites, such as peroxynitrite and H_2_O_2_,^17,18^ CO_2_ influences posttranslational modification of proteins through carbamylation^47,48^ and influences gene expression.^49,50^ The participation of CO_2_ in the modulation of cell physiology and pathology by a series of mechanisms emphasizes the urgency of more studies of the effects of CO_2_ on mammalian cell metabolism.^18^

## Supporting Information Available

Fluorescence microscopy, including experimental conditions and results; detection of Fl in resting and in PMA-activated macrophages in the presence of Fl-B (Figure S1); effect of catalase on Fl detection in resting macrophages (Figure S2) (PDF)

## Abbreviations

Amplex Red: 10-acetyl-3,7-dihydroxyphenoxazine
CBE: coumarin-7-boronic acid pinacolate ester
COH: 7-hydroxycoumarin
DPBSG: Na_2_HPO_4_ (21.3 mM), KH_2_PO_4_ (3.87 mM), KCl (2.67 mM), NaCl (138 mM), MgCl_2_ (0.49 mM), CaCl_2_ (0.88 mM), glucose (5.5 mM), and DTPA (0.1 mM), pH 7.4, containing 0, 21.6, or 42.2 mM HCO_3_^–^ equilibrated with 0, 5, or 10% CO_2_, respectively
DTPA: diethylenetriaminepentaacetic acid
Fl: fluorescein
Fl-B: fluorescein-boronate
HRP: horseradish peroxidase
SOD: superoxide dismutase
peroxynitrite: sum of peroxynitrite anion (ONOO^–^) and peroxynitrous acid (ONOOH)
PMA: phorbol 12- myristate 13-acetate
