# Supplementary material for: Production of Peroxymonocarbonate by Steady-State Micromolar H2O2 and Activated Macrophages in the Presence of CO2/HCO3– Evidenced by Boronate Probes
Source: Chem Res Toxicol. 2024 Jun 25;37(7):1129–38. doi: 10.1021/acs.chemrestox.4c00059 (PMC11256887; doi:10.1021/acs.chemrestox.4c00059)
Supplement: Supplementary file 1 — tx4c00059_si_001.pdf [file tx4c00059_si_001.pdf]

Supporting Information for

"Production of peroxymonocarbonate by steady-state micromolar H_2O_2 and activated macrophages in the presence of CO_2/HCO_3^- evidenced by boronate probes"

Edlaine Linares[†], *Divinomar Severino*[†], *Daniela R. Truzzi*[†], *Natalia Rios*[‡§], *Rafael Radi*[‡§], *Ohara Augusto*[†]*

[†]Departamento de Bioquímica, Instituto de Química, Universidade de São Paulo, Brazil and [‡]Departamento de Bioquímica and [§]Centro de Investigaciones Biomédicas (CEINBIO), Facultad de Medicina, Universidad de la República, Montevideo, Uruguay

* To whom correspondence should be addressed: E-mail: oaugusto@iq.usp.br

Table of contents

Title page……………………………………………………………………………….S1

Table of contents………………………………………………………………………..S2

Fluorescence microscopy: Experimental conditions..…………………………………..S3

Fluorescence microscopy: Results……………………………………..…………….S4

References…………………………………………………………………………...S7

Figure S1. Detection of Fl in resting and in PMA-activated macrophages in the presence of Fl-B by fluorescence microscopy…………………………………………………S8

Figure S2. Effect of catalase on Fl detection in resting macrophages…………………S9

Fluorescence microscopy: Experimental conditions

Raw macrophage cells (264.7 cell line) were obtained and cultured as previously described.[1] Before treatments, cells (5 x 10^4 cells/well) were plated in 96-well plates and maintained under the same conditions for 72 h. Confluent cells washed three times received DPBSG containing 0, 21.6 or 42.2 mM HCO_3^- equilibrated with 0, 5 or 10% CO_2, respectively.[1] Then, the cells were incubated with 30 μM Fl-B for 10 min at 37 ºC in a microplate reader maintained at 0, 5 or 10% CO_2 before addition or not of PMA (1 μg/mL) (total volume of 200 μL). After 1 h incubation, cells were washed and analyzed in PBS by fluorescence microscopy in a customized Edinburgh Instruments Fluorometer FLS 920 coupled to an Eclipse Ti Nikon microscope. The system consisted of Xenon bulb lamp 450 W housing an excitation monochromator and a fiber guide to excite the sample through a 10x objective. The images were obtained by a Nikon Digital Sight DS-Qi1Mc monochromatic camera. The filters inside the cube were Nikon 777074524 T495 Ipxr C173798, which contain only a dichroic mirror reflecting light lower than 495 nm, allowing sample excitation at 470 nm and getting fluorescence images emitting above 495 nm, and an additional long pass filter 495 nm was placed after cube. The fluorescence intensity values of the microscopic images were obtained by measuring the fluorescence integral density using ImageJ Fiji Software. The fluorescence intensity values were normalized by the average value of the Fl-B group of each independent experiment. The data were plotted and analyzed statistically (t-test) with the GraphPad Prism software.

Fluorescence microscopy: Results

Confluent macrophages in modified DPBSG in the absence or presence of CO_2/HCO_3^- were incubated with 30 µM Fl-B for 10 min at 37 ºC in a microplate reader maintained at 0, 5 or 10% CO_2 before addition or not of PMA (1 µg/mL). After 1h incubation, washed cells were transferred to PBS and analyzed by fluorescence microscopy. Representative images of the macrophages after incubation at each specified condition showed the presence of oxidized Fl-B localized in the cells, either intracellularly and/or bounded to cellular membranes (Figure 1S). This figure also shows the normalized relative values of the fluorescence intensity of the images obtained in triplicates of three independent experiments at each condition and the corresponding average values ± SE.

PMA-activated macrophages presented average levels of cellular fluorescence (Figure 1Sb, red dots) higher than resting macrophages (Figure 1Sb, blue points) both in the absence and presence of CO_2/HCO_3^-. In the case of activated cells, however, no difference was noticeable between the tested concentrations of CO_2/HCO_3^-, in contrast with the experiments following total fluorescence of the bulk solution (extra- plus intracellular) (Figure 5 in ref.[1]). Such discrepancy may be due to the change of the environment of the cells before observation in the microscopy experiments (from 19.8 or 18.2% O_2 and 5 or 10% CO_2, respectively to ambient atmosphere). This change also likely contributed for the considerable variation observed among the experiments, particularly in the presence of CO_2/HCO_3^- (Figure 1S). In addition, PMA-activated RAW macrophages produce H_2O_2 extracellularly, slowly and in low concentrations under our experimental conditions (Figure 3 in ref.[1]). This limits the concentrations of H_2O_2 that can be attained intracellularly (around 1% of the extracellular concentration)[2] and, consequently, of its product HCO_4^-.[3] As a result, it is likely that the fluorescence observed

in PMA-activated macrophages primarily originated from the extracellular oxidation of Fl-B, with minimal contributions from intracellular oxidation.

Resting macrophages showed considerably lower levels of fluorescence intensity than activated cells, suggesting the possibility of Fl-B oxidation by H_2O_2 formed during cell metabolism.[2,4] In the presence of CO_2/HCO_3^-, the average fluorescence intensity increased and tended to be dependent on CO_2/HCO_3^- level (Figure 1S, blue dots). These results suggested that Fl-B could be able to detect HCO_4^- formation from low basal levels of H_2O_2. However, additional experiments adding catalase (250 U/mL) to the media caused a strong, but not complete inhibition of the fluorescence of the cells in the absence and presence of CO_2/HCO_3^- (Figure S2). These experiments suggest that a fraction of Fl-B can be oxidized intracellularly by resting macrophages, but the probe is not sensitive enough to distinguish basal H_2O_2 from the HCO_4^- eventually formed from it. Alternatively, the background fluorescence observed in the presence of catalase could be due to the limited hydrolysis of Fl-B to Fl in aqueous solutions (approximately 1%),[5] followed by Fl entry into cells.

In conclusion, the Fl-B fluorescence microscopy results did not expand the data presented in our work[1], but provided useful clues for future studies of HCO_4^- formation in cells. Control of the cell atmosphere during cell microscopy and cell sorting experiments is critical, as is the selection of the appropriate cell system and boronate probe. PMA-activated macrophages slowly produced low levels of extracellular H_2O_2 under our experimental conditions,[1] limiting the eventual levels of intracellular oxidants. Furthermore, although the predominant species of Fl is the dianionic form, it is in equilibrium with small proportions of the monoanionic and neutral forms.[5,6] In long incubations such as those required here,[1] the neutral form that occurs intracellularly or

extracellularly can eventually traverse cell membranes. The knowledge gained from these preliminary results allow design of possible future experiments.[1]

References

(1) Linares, E.; Severino, D.; Truzzi, D. R.; Rios, N.; Radi, R.; Augusto, O. Production of peroxymonocarbonate by steady-state H_2O_2 and activated macrophages in the presence of CO_2/HCO_3^- evidenced by boronate probes, *Chem. Res. Toxicol.* 2024, 10.1021/acs.chemrestox.4c00059.

(2) Sies, H.; Jones, D. P. Reactive Oxygen Species (ROS) as Pleiotropic Physiological Signalling Agents. *Nat. Rev. Mol. Cell Biol.* **2020**, *21* (7), 363–383. https://doi.org/10.1038/s41580-020-0230-3.

(3) Truzzi, D.R.; Augusto, O. Influence of CO2 on Hydroperoxide Metabolism. In *Hydrogen peroxide in Health and Disease*; Vissers, M.C.M., Hampton, M., & Kettle, A.J. (Eds.). (2017). Hydrogen Peroxide Metabolism in Health and Disease (1st ed.). CRC Press. https://doi.org/10.1201/9781315154831: Boca Raton, FL, 2017; pp 83–101.

(4) Augusto, O.; Truzzi, D. R. Carbon Dioxide Redox Metabolites in Oxidative Eustress and Oxidative Distress. *Biophys. Rev.* **2021**, *13* (6), 889–891. https://doi.org/10.1007/s12551-021-00860-3.

(5) Rios, N.; Piacenza, L.; Trujillo, M.; Martínez, A.; Demicheli, V.; Prolo, C.; Álvarez, M. N.; López, G. V.; Radi, R. Sensitive Detection and Estimation of Cell-Derived Peroxynitrite Fluxes Using Fluorescein-Boronate. *Free Radic. Biol. Med.* **2016**, *101*, 284–295. https://doi.org/10.1016/j.freeradbiomed.2016.08.033.

(6) Sjöback, R.; Nygren, J.; Kubista, M. Absorption and Fluorescence Properties of Fluorescein. *Spectrochim. Acta A: Mol. Biomol. Spectrosc.* **1995**, *51* (6), L7–L21. https://doi.org/10.1016/0584-8539(95)01421-P.

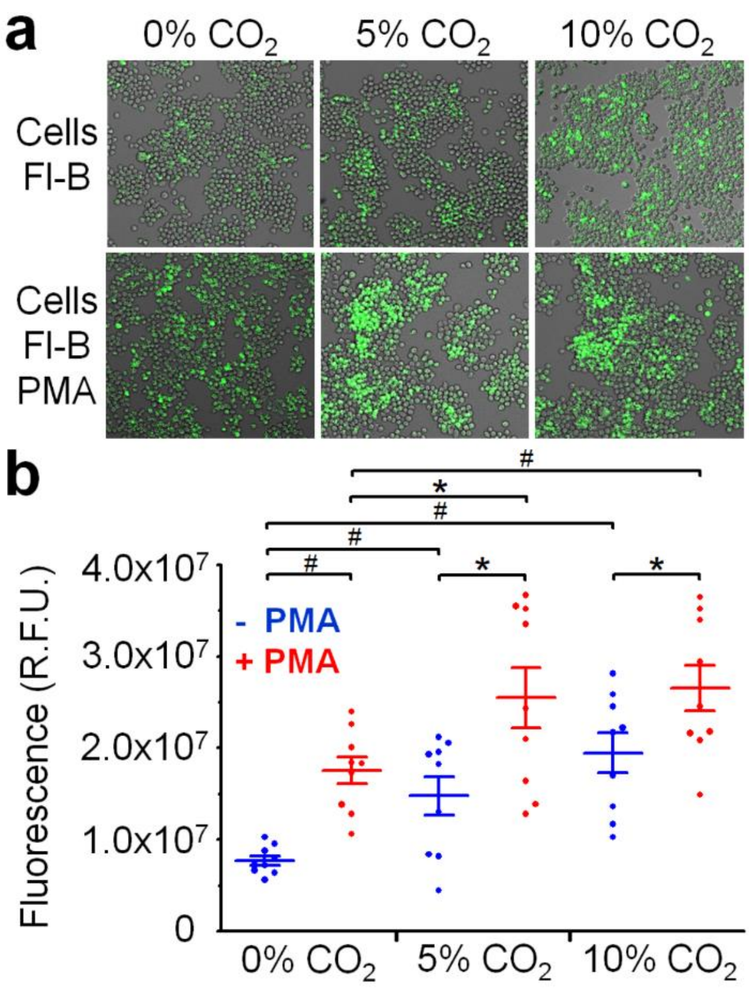

Figure 1S. Detection of Fl in resting and in PMA-activated macrophages in the presence of Fl-B. Confluent cells in 96-well plates in DPBSG containing 30 μM Fl-B in the absence and presence of CO_2/HCO_3^- at the specified concentrations were incubated for 10 min at 37 ºC before activation or not with PMA (1 μg/mL). After 60 min, washed cells transferred to PBS were examined by fluorescence microscopy. (a) Representative images of intracellular Fl-B oxidation at each condition. (b) Mean relative fluorescence intensity values ± S.E. from three repetitions of three independent experiments at each condition (t-test, # ≤ 0.01, * ≤ 0.05).

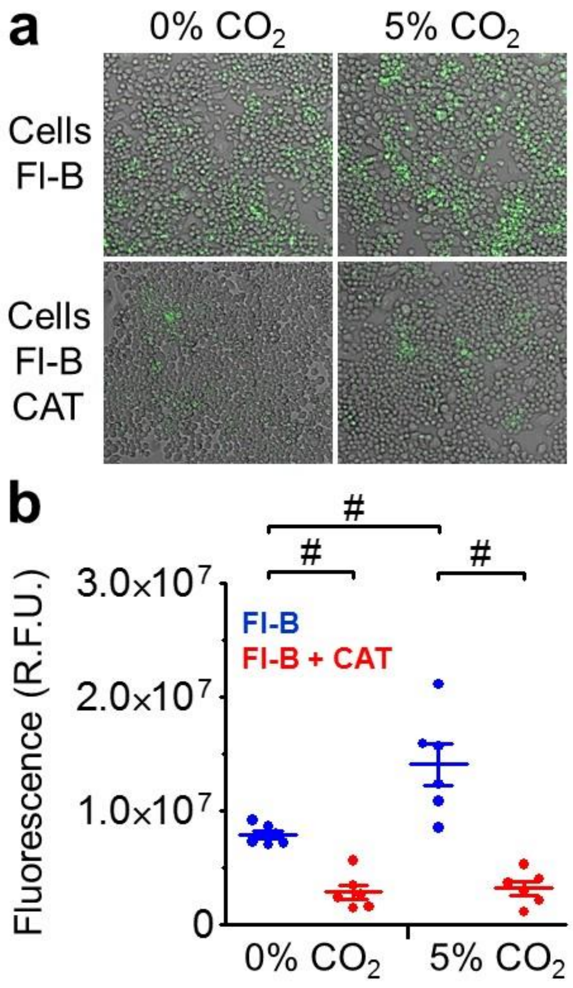

Figure S2. Effect of catalase on detection of Fl in resting RAW macrophages in the presence of Fl-B. Confluent cells in 96-well plates in DPBSG containing 30 μM Fl-B in the absence and presence of catalase (250 U/mL) and CO_2/HCO_3^- (5%/21.6 mM) were incubated for 60 min at 37 ºC. Then, cells were washed, transferred to PBS and examined by fluorescence microscopy. (a) Representative images of intracellular Fl-B oxidation at each condition. (b) Mean relative fluorescence intensity values ± S.E. from three repetitions of two independent experiments at each condition (t-test, # ≤ 0.01).
